# Medical education in times of war: a mixed-methods needs analysis at Ukrainian medical schools

**DOI:** 10.1186/s12909-023-04768-2

**Published:** 2023-10-26

**Authors:** Anja Mayer, Olena Yaremko, Tetiana Shchudrova, Olena Korotun, Karolin Dospil, Inga Hege

**Affiliations:** 1 Medical Education Sciences, University of Augsburg, Universitätsstraße 2, 86159 Augsburg, Germany; 2 Educational Department, Bukovinian State Medical University, Teatralna Sq. 2, Chernivtsi, 58002 Ukraine; 3 Department of Pediatrics and Children Infectious Diseases, Bukovinian State Medical University, Teatralna Sq. 2, Chernivtsi, 58002 Ukraine; 4 Institute for Medical Education, LMU Klinikum Munich, Pettenkoferstraße 8a, 80336 Munich, Germany

**Keywords:** Ukraine, War, Medical education, Needs, Qualitative research

## Abstract

**Background:**

As Ukraine struggles with the education of healthcare professionals due to the war, we aimed to identify the specific effects of the war on medical education, the resulting needs, and the expected consequences for schools, faculty, staff, students, and the healthcare system.

**Methods:**

In October and November 2022, we performed a survey of students, faculty, and staff of medical schools in Ukraine and conducted semi-structured interviews with faculty leaders (i.e., rectors, vice-rectors). We conducted a descriptive analysis of the survey’s closed-ended questions. The survey and the interviews included open-ended questions about war-related restrictions to teaching and learning, resulting needs, and expected consequences, for which we applied a thematic analysis.

**Results:**

We received 239 survey responses (*N* = 49 faculty and staff, *N* = 190 students) and conducted nine interviews with faculty leaders across Ukraine. Most survey participants indicated that they had experienced restrictions or changes to their work or study due to the war (86% of faculty and staff, 69% of students). From the thematic analysis of the survey and interviews, we identified eight themes: disruption of teaching, increased workload, mental stress, financial restrictions, non-war related needs, international cooperation, quality of education, and prospects of future professionals. The quality of healthcare education in Ukraine was threatened, and schools, faculty, staff, and students were under great strain. While already established international cooperation has been supportive, some needs have still not been addressed.

**Conclusions:**

We hope that our findings will help researchers and educators from abroad contribute to meeting Ukraine’s needs in medical education.

**Supplementary Information:**

The online version contains supplementary material available at 10.1186/s12909-023-04768-2.

## Background

### Medical education in Ukraine

Medical education in Ukraine has been considered “both one of the highest quality and relative affordability globally” [1]. About 70,000 students (69% female, 31% female) [2, 3] are studying at 36 medical schools [4], consisting of universities and academies located in 19 cities across the country. Also, before the war, Ukraine hosted more than 26,000 international medical students, mostly from India, Africa, and the Middle East [5]. In 2005, Ukraine entered the Bologna Process which aims to harmonize the various systems of higher education in Europe, by implementing a system of quality assessment, mutual recognition of qualifications, and the introduction of bachelor’s and master’s study programs [6]. As a result, the standard period of study for a medical degree in Ukraine is 6 years, consisting of a 4-year bachelor’s program and a 2-year master’s program [7]. However, there are still challenges slowing down the reforms [8, 9]. For example, most medical schools in Ukraine are not part of a university hospital and rely on contracts with nearby hospitals [10], resulting in limited patient contact. However, promising changes have been achieved, such as the implementation of the nationwide Unified State Qualification Exam (USQE) in 2019 [11, 12]. Also, the digitization of medical education was accelerated by the COVID-19 pandemic and is well underway [13].

### Current situation in Ukraine

On February 24th, 2022, these developments were stopped when the Russian Federation launched an armed offensive. Since that day, Russia’s army has destroyed military facilities, cities, and critical infrastructure across Ukraine [14]. Civilians and soldiers are suffering physical and mental injuries [15, 16] and access to humanitarian aid and healthcare is limited due to a lack of healthcare professionals, insufficient medical supplies, and the destruction of healthcare facilities [16]. Consequently, incidences of war-related conditions, such as injuries, psychological traumata, or infectious diseases are rising, and the management of chronic diseases will probably worsen [17, 18].

Armed conflicts affect the content and quality of medical education [19] and students in Ukraine may delay their studies, or not graduate, or miss out on key learning objectives [1, 16]. For example, students and educators may need to flee from the war or serve at the frontlines [15, 20]. Furthermore, the lack of practical skills that Ukrainian graduates showed before the war, due to remote and hybrid teaching during the COVID-19 pandemic, has aggravated the problem [1, 13].

### Needs of medical education in time of war

In their scoping review of maintaining healthcare education during times of war, Dobiesz et al. identified five categories: curriculum, personnel, wellness, resources, and oversight. They described barriers such as threat to safety, decrease in the number of students and teachers, and loss of resources. They suggested targeted interventions such as structural curricular changes and the use of online resources [21].

Duration, localization, and intensity of a war strongly influence the needs and prospects of a country’s medical education. Long-lasting wars covering large areas and costing thousands of lives, such as in Afghanistan, Liberia, and Iraq, can severely affect the quality of medical education [22–24]. However, a study of students who had provided medical support in the locally confined one-month Lebanon-Israel war showed that they felt more compassionate towards their patients, were prouder of their profession, and were more experienced in emergency medicine [25].

Students, faculty, staff, and faculty leaders have different perspectives and needs during a war. A study of the mental well-being of medical students during the war in Syria showed that especially low-income students suffered from depression, stress, and anxiety. The authors of that study recommended psychological as well as financial programs to support students [19]. Lafta et al. investigated the long-term effect of personal and professional circumstances of the war on medical students in Iraq, finding that most students were dissatisfied with the quality of education and poor career prospects, and willing to leave the country after graduation. The authors recommended using modern teaching methods, accelerating digitization, and “sustain[ing] the motivation that originally attracted students to study medicine” [26].

Clinical educators are or feel obliged to provide as much medical care as possible while maintaining high-quality teaching for students [27].

In their review of challenges to the education of health professionals during the war in Syria, Bdaiwi et al. argue that education should be seen in the larger context of the healthcare system and organizational and programmatic aspects. They recommended a locally driven education strategy, with increased funding, regulatory structures, and international collaboration [28].

Although the situation and needs of medical students during a war has been explored in various studies, the perspective of faculty members and staff is less evident. Also, the needs of medical education in times of war differ according to the circumstances of the war and are influenced by recent developments in technology and events such as the COVID-19 pandemic. Therefore, the aim of this study is to identify the specific effects of the Russia-Ukraine war on faculty, staff, faculty leaders, and students in Ukraine, the needs that result from it, and the expected consequences.

## Methods

To answer this research question, we used a mixed-methods approach. We implemented a survey among faculty, staff, and students to understand their perspectives, and conducted semi-structured interviews with faculty leaders (rectors, vice-rectors, heads of departments), to gain an in-depth understanding of the schools’ situation and needs. For reporting the results of the thematic analysis, we followed the Consolidated Criteria for Reporting Qualitative Studies (COREQ) recommended by Tong et al. [29]. The study was approved by the Institutional Review Board of the Ludwig-Maximilians-University, Munich, Germany (No. 22–0726).

### Development of the survey and interview guideline

All authors developed the survey questions jointly, based on a survey assessing the need for teaching clinical reasoning among medical students and faculty [30, 31], following the best practices for item-writing recommended by Artino et al. [32]. The survey encompassed personal data, three single-choice and eight open-ended questions about war-related restrictions to teaching and study, resulting needs, measures taken to handle the situation, and expected impact of the war on teaching, students’ future professional life, and the healthcare system. To assess how familiar teachers and students are with forms and methods of remote teaching, we added a multiple-choice question. We created separate versions of the survey for students and faculty, adapting the questions to their perspectives (see Additional file 1: Appendix 1). We implemented them using the LimeSurvey platform, with a tick box on the starting page to give consent for data use. The survey was anonymous and available in English and Ukrainian. We piloted the survey with 12 Ukrainian and German students, faculty, and staff members two weeks before the study to assess the appropriateness of the content and questions. While the survey was well received and did not lack any important aspects, we changed the wording of two questions to enhance understandability.

For the interview guideline, we adapted the open-ended questions of the survey to the perspective of faculty leaders and added a question about international cooperation and policy development (see Additional file 1: Appendix 2).

### Data collection

On October 10^th^, 2022, we sent out an email to medical schools across Ukraine listed in the World Directory of Medical Schools [4] and to the International Federation of Medical Students Association Europe [33]. The email included study information, a link to the online survey, and a request to forward the email to faculty, staff, and students. We sent a reminder after two weeks and closed the survey after six weeks.

At the same time, we identified faculty leaders on the schools’ websites and asked them via email to have a semi-structured interview with a psychologist (OY) on the video conferencing platform Zoom. Based on our pilot test we set a time of 30 min. for the interviews. We used a purposive sampling strategy aimed at including interviewees from different parts of the country. Prior to the interview, participants gave their written consent. We conducted interviews until data saturation was reached, i.e., we stopped recruitment when we saw that no more new themes emerged from the data.

### Analysis

Personal data and answers to closed-ended questions of the survey were analyzed using descriptive statistics of SPSS software (version 29.0). We translated answers to open-ended questions into English, and coded them using MAXQDA software, applying the coding framework we had previously developed when analyzing the interviews (see below).

The interviews were audio-recorded, transcribed verbatim, anonymized, and translated into English. Two researchers (OY, AM) carried out a thematic analysis of the transcripts following the six steps for qualitative content analysis suggested by Kuckartz [34]. Using an inductive approach, they independently identified codes for a subset of two interviews and reached consensus on a first coding framework. They then coded one interview after another, applying the coding framework and developing it further in an iterative process. They used MAXQDA software (version Analytics Pro 2000) for the coding and discussed discrepancies until consensus was reached. OY, IH, and AM then grouped similar codes into themes. Throughout the process KD, OK, and TS reviewed the coding framework and emerging themes and provided feedback; discrepancies were discussed until consensus was reached.

## Results

### Participants

#### Interviews

**Fig. 1:**
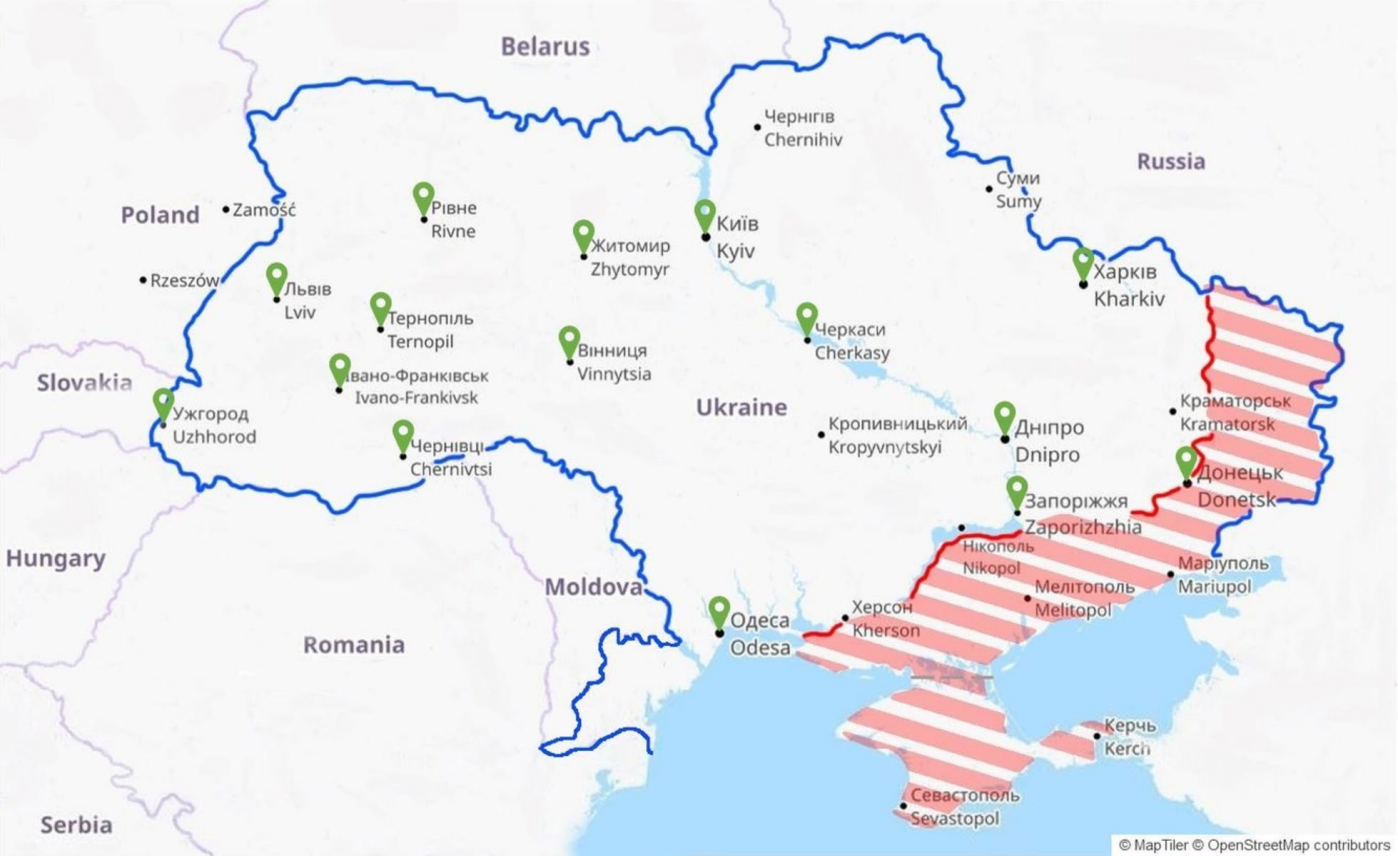
Geographical location of cities of participants’ medical schools. This image was generated using OpenStreetMaps. The occupied zone (marked in red) is shown as it was on October 10^th^, 2022 [35]. For further details, see Additional file 1: Appendix 3

We conducted nine interviews with faculty leaders (*N* = 3 female, *N* = 6 male) in nine cities across Ukraine, one of which was in the occupied zone (see Fig. 1).

#### Survey

We received 239 responses from 49 faculty members and 190 students (see Table 1) working or studying at Ukrainian medical schools. Participants’ schools (*N* = 19) were located in 13 cities across Ukraine (see Fig. 1 and Additional file 1: Appendix 3), but we did not receive any responses from schools in the occupied zone. The average age was 47 years for faculty and staff (range: 28–70), and 20 years for students (range: 16–45). 71% of participants were female.

**Table 1:** Description of survey participants

Variable	Description	Faculty (*N* = 49)		Students (*N* = 190)	
		**[N]**	**[%]**	**[N]**	**[%]**
Age	Mean (range)	47 (28–70)		20 (16–45)	
Sex	Female	35	71%	135	71%
	Male	14	29%	55	29%
Nationality	Ukraine	47	96%	184	97%
	Other	2	4%	3	2%
	Missing	0	0%	3	2%
Educational program mostly related to	Medicine	45	92%	182	96%
	Pediatrics^a^	4	8%	8	4%
Years of working experience	Mean (range)	16 (1–47)		-	-
Primary role(s)	Teacher of clinical department	27	55%	-	-
	Teacher of basic sciences	18	37%	-	-
	Head of department	4	8%	-	-
	(Deputy) dean	3	6%	-	-
	Administration	3	6%	-	-
	Other	1	2%	-	-
Educational level/ Academic title	Ph.D./Doctoral degree	25	51%	-	-
	Postdoc	9	18%	-	-
	Associate/Assistant Professor	21	43%	-	-
	Professor	9	18%	-	-
	Other	6	12%	-	-
Year of study	1	-	-	59	31%
	2	-	-	22	12%
	3	-	-	38	20%
	4	-	-	19	10%
	5	-	-	34	18%
	6	-	-	10	5%
	Intern	-	-	3	2%
	Missing	-	-	5	3%
Educational level applied for	Master^b^	-	-	165	87%
	Bachelor	-	-	8	4%
	Other or missing	-	-	17	9%
Primary role(s)	Budget student (financed by the state)	-	-	149	78%
	Contract student (self-payment)	-	-	38	20%
	Missing	-	-	3	2%

^a^In Ukraine, Pediatrics is a separate study program, ^b^Most Ukrainian medical students, who start with the bachelor's program, plan to pursue a master’s degree

51% of faculty and staff reported having a Ph.D./doctoral degree. 43% described themselves as being assistant or associate professors, 18% professors and 18% postdocs. Concerning their primary role, 55% indicated being a clinical teacher, 37% teacher of basic sciences, 8% head of department, 6% dean or deputy dean, and 6% administrative staff.

Students from all years of study participated in the survey, with the majority (73%) being in year 1–4 and 23% in year 5 or 6.

### Results of single-choice questions related to war and multiple-choice questions on remote teaching

About 35% of faculty and staff and 15% of students reported that their region had been a zone of active fighting since the beginning of the war (Table 2). About 20% of the participants had been forced to flee.

**Table 2:** Questions related to war and remote teaching

Variable	Description	Faculty (*N* = 49)		Students (*N* = 190)	
		**[N]**	**[%]**	**[N]**	**[%]**
Region of school has been an active combat zone	Yes	17	35%	29	15%
	No	29	59%	112	59%
	Missing	3	6%	49	26%
Had to flee from home because of the war	Yes	10	20%	39	21%
	No	36	73%	105	55%
	Missing	3	6%	46	24%
Restrictions or changes to work/study due to the war (faculty and staff/students)	Yes	42	86%	131	69%
	No	7	14%	42	22%
	Missing	0	0%	17	9%
Remote teaching formats used/participated in	Lecture	33	67%	119	63%
	Workshop	35	71%	124	65%
	Seminar	22	45%	75	39%
	Case-based learning	24	49%	40	21%
	Problem-based learning	15	31%	7	4%
	Learning-Management-Systems	24	49%	65	34%
	Screencasts /videos	25	51%	45	24%
	Game-based learning	11	22%	27	14%
	Virtual Patients	11	22%	13	7%
	Other	1	2%	3	2%
	None	0	0%	1	1%
	Missing	10	20%	48	25%

Most participants (86% of the faculty and staff, 69% of the students) reported that their work or study had been restricted or changed because of the war.

When asked for remote teaching formats, most faculty members and students reported experience with lectures (67% and 63%) and workshops (71% and 65%), many also being familiar with learning-management-systems (49% and 34%) and screencasts (51% and 24%).

### Thematic analysis of interviews and open-ended survey questions^1^

**Fig. 2:**
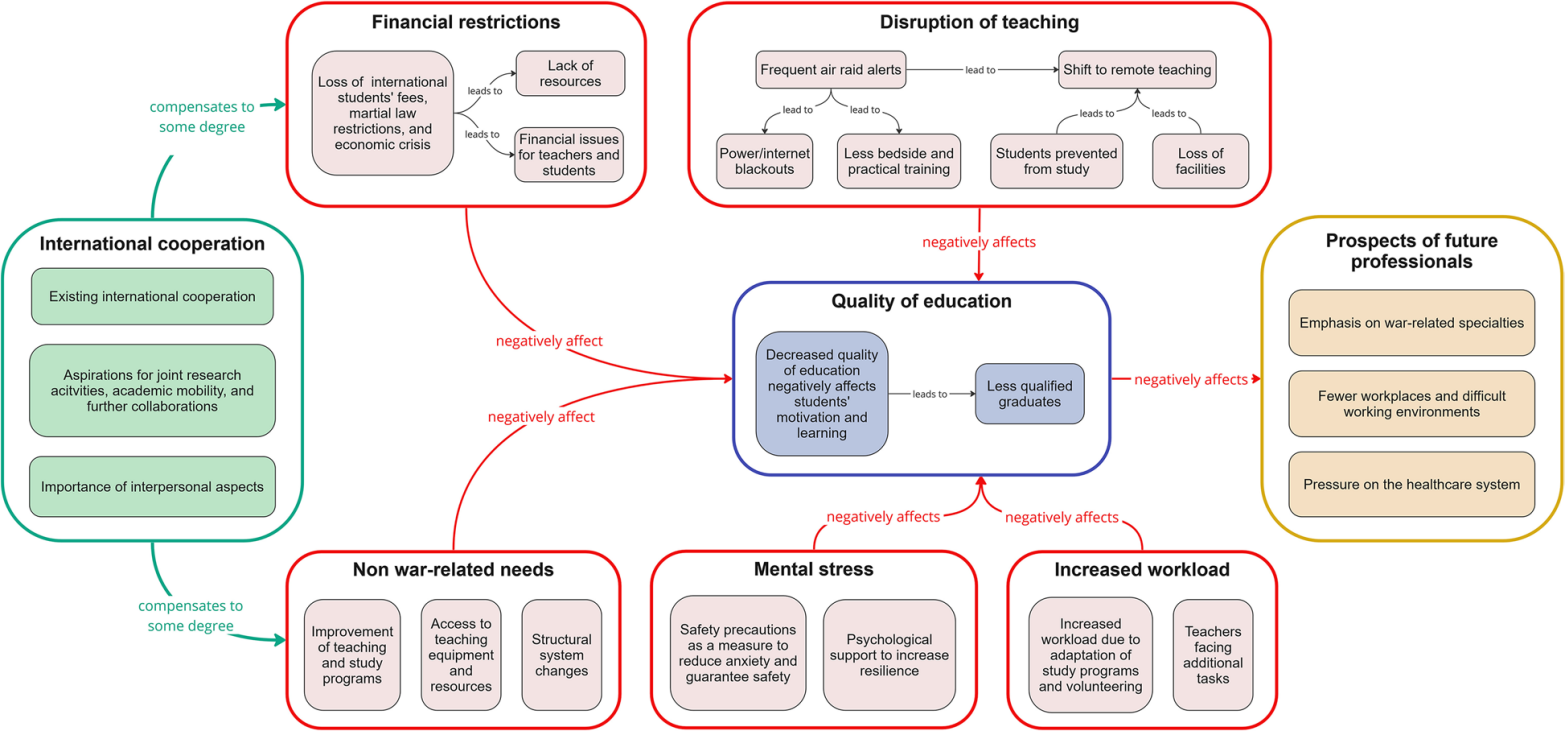
Graphical representation of themes derived from thematic analysis

Most participants agreed that they *“need the war to end as soon as possible” (S78_S)* and *“peace is the main remedy for the situation” (S148_F).* Beyond that, we identified the following eight themes (see Fig. 2):

- Disruption of teaching
- Increased workload
- Mental stress
- Financial restrictions
- Non-war related needs
- International cooperation
- Quality of education
- Prospects of future professionals

#### Disruption of teaching

The disruption of teaching had a strong negative impact on medical education in Ukraine, mainly due to (a) students’ being prevented from study, (b) loss of facilities, and frequent air raids resulting in (c) less bedside teaching and practical training, and (d) frequent power and internet blackouts. To maintain teaching, schools (e) implemented remote teaching formats.

Participants reported that at the beginning of the war, many Ukrainian and most international students had gone to western cities or abroad, experiencing some interruption in their studies. For example, one international student at Kharkiv University, who fled when the war broke out, reported: “*The course is online now, but also being offered offline in Western Ukraine (Ivano-Frankivsk), but I think it's still unsafe to travel back to Ukraine, unless there's ceasefire, and peace prevails again” (S141_S)*. Some international students even had to quit their studies *“because our home country's national medical board declared that it's not going to recognize our ‘online education’” (S138_S)*. Although many Ukrainian students returned later, there was still migration from the more affected regions to western or centrally located cities in Ukraine. Schools *“took in […] students and graduate students” (I10)* but soon reached their capacity. Therefore, *“part of the [Ukrainian] students have simply dropped out, a minority has gone abroad, who might […] have found an opportunity to continue studying at other universities” (I3). “Most of those we have lost, however, were international students, because they could not come, or could not afford it any longer, or had other reasons” (I6).* Other students experienced difficulties in continuing their studies because they were serving on the front lines or living in an occupied zone where teaching was continued remotely, often with *“an individual study schedule” (S171_S).*

Relocation of schools to a safe location, or destruction of facilities, led to a disruption of teaching with “*difficult inventory and logistics management circumstances, because we were not given the chance to evacuate the equipment that we had” (I6).*

Participants emphasized that the *“almost daily shelling of Ukrainian cities” (S281_S)* caused a major disruption in teaching, endangered students, faculty, and staff on their way to school, and forced them to stay *“in a shelter (…) during air raid alerts, and as a result, the inability to conduct classes in full” (S194_F)*. While students close to the front lines experienced some patient contact and practical training by supporting the care for injured civilians and soldiers, others lacked bedside teaching because many hospitals did not provide sufficient shelters, students were not allowed to enter simulation centers *“due to safety risks” (I5)* or interrupted practical classes could not be continued in the shelters. Facilities for practical training were generally scarce, so “*groups that missed their classes (…) might overlap [with other groups]” (I3)*.

Due to missile attacks targeted at infrastructure, power and internet blackouts led to disruption of both online and face-to-face teaching *“as electricity might be unexpectedly switched off or on, and all this affects (…) the learning and communication means” (I9).* As a result, one of the main needs was *“a stable energy system” (I9).*

At the beginning of the war, schools across the country had switched to remote or hybrid teaching for security reasons and to continue teaching. Due to the experience during the COVID-19 pandemic, schools were already *“adjusted to online education” (I10).* Depending on the situation, schools were able to return to face-to-face teaching but *“were forced to switch [back] to remote teaching due to the threat of missile attacks” (S132_F)*.

#### Increased workload

Participants suffered from an increased workload due to (a) adaptation of study programs and (b) volunteering, where (c) teachers faced additional tasks.

Adaptations of study programs not only included earlier starts of semesters, but also *“shortened term of study” (S145_S)* by introducing *“classes on Saturdays” (S86_S)* or *“tightening of curricula” (S146_S).* Also, schools introduced teaching in shifts, depending on *“how many [students] could stay in the facilities depending on the capacities of the shelters” (I1).* These adaptations of the study program led to an increased workload for students, teachers, and *“administration [who] creates individual schedules of classes” (S194_F).*

In case teaching was disrupted by air raid alerts, students and teachers either *“had to put in extra hours, to catch up, to get the work done” (I10)* or students were responsible for filling the gap on their own with more *“independent work and self-study” (S26_F).* Thus, students expressed their need for a *“reduced workload” (S41_S)* and participants expected that *“part of [them] will lose the quality of education, primarily those students who are [not used to] educating themselves” (I9).*

Students and faculty near the frontlines volunteered in *“first-zone medical facilit[ies]” (I7)*, whereas in less affected regions they supported refugees *“with serious health conditions, with chronic illnesses, or acute conditions” (I10)*. Also, teachers trained soldiers and citizens *“to provide first aid [or] taught them how to stop bleeding” (I3)* in simulation centers. One school even launched *“a psychological rehabilitation center where we offer advice to [relocated] patients [and]a call center assisting everyone in need” (I8).*

Overall, the loss of teachers who migrated, volunteered at the frontlines, or were dismissed due to budget restrictions, led to an *“increased workload for those who remained” (S132_F)*. Also, from the beginning of the war, many teachers had been *“active in additional international projects, which increase[d] the workload and the risk of professional burnout” (I5).* Therefore, *“better working conditions to prevent the outflow of qualified teachers” (S132_F)* were needed.

#### Mental stress

This theme includes (a) anxiety, (b) safety precautions, (c) psychological support, and (d) resilience.

Faculty and students suffered from mental stress and anxiety with a negative effect on students’ learning: *“In the days when air raids are long, and the phone has an endless stream of messages about missile strikes on the cities of your country, it is extremely difficult to study, the emotions that you experience are tiring” (S21_S)*. Even in the absence of air raid alerts, students reported that *“it became harder to perceive information and focus on the learning process” (S19_S),* leading to *“poor academic performance” (S207_S).* Teachers expected that the *“excessive levels of stress and anxiety negatively affect the psychological state [of students], which in the future will lead to an increase in psychological and mental disorders among graduates” (S233_F).*

Safety precautions to reduce anxiety and guarantee safety of faculty, staff, and students in case of air raids included making evacuation plans and providing shelters nearby. However, possibilities were limited, and at some schools there still was need for improvement in the amount, quality, and proximity of shelters.

To foster resilience and prevent negative consequences of mental stress and anxiety, schools offered *“psychological support for both our teachers and our students” (I5).* Despite the difficult circumstances, interviewees thought that the Ukrainians *“adapted for the reality that we are currently in” (I8)* or even *“that it will make us stronger” (I9).*

#### Financial restrictions

There were financial restrictions due to (a) the loss of international students and (b) martial law restrictions leading to (c) lack of resources and (d) financial issues for teachers and students. The situation was worsened by (e) the economic crisis due to the war.

The *“decrease in the number of foreign students” (S135_F)* resulted in a loss of tuition fees for schools. To counteract this, participants suggested *“a special transit visa [allowing them] to fly through a country of the European Union and reach the border of Ukraine” (I9).*

*“Due to the martial law restrictions”* which allowed schools to spend their money only on the bare necessities, schools were limited in buying teaching equipment, such as *“books, (…) literature, and (…) mannequins” (I3)*. Budgetary constraints also resulted in postponing repair of buildings and *“reducing salaries” (S26_F*) for teachers, who asked for *“support from the state” (S51_F)*.

The economic crisis worsened the situation of teachers and "*parents that pay tuition fees” (F148_F)* as *“inflation effectively reduced wages by half” (I4)*.

#### Non war-related needs

General needs mentioned by participants were (a) improvement of teaching and study programs, (b) access to teaching equipment and resources, and (c) structural system changes.

Aside from the war, students and faculty wished for *“a completely different approach to teaching” (S175_S)* and suggested *“re-training of teaching staff” (S200_F).* They suggested improving study programs by integrating foreign languages, *“updat[ing] the curriculum” (S63_S),* and *“increas[ing] the time of practical classes and visits to patients” (S277_S)*. Accordingly, participants stressed that additional university hospitals are a *“prerequisite for quality training of doctors” (S247_F).*

There was a need for more or better teaching equipment, such as *“equipment for simulation centers” (S247_F)* or *“modern laboratories” (S25_F*) and a need for technical infrastructure and devices such as “*large databases of (…) teaching materials*” *(S265_S)* and *“simulation training [and] virtual patients” (S16_F).*

Faculty and students wanted changes in processes and structures within their schools including *“less bureaucracy in state institutions” (S211_S)*, *“eradication of corruption” (S3_S)*, *“valuing students as equal participants in the educational process [and] abandoning the Soviet hierarchical model in education” (S15_F).*

#### International cooperation

This theme covers (a) existing international collaborations, (b) aspirations for joint research activities, academic mobility, and further collaborations, and (c) the importance of interpersonal aspects in international cooperation.

Faculty leaders hope that international collaborations, which had been established after the beginning of the war and led to the provision of generators or train-the-trainer courses, will continue in the future. Also, they expressed their need for further support, *“for the purchase of medical equipment, training equipment like mannequins, or funding for the renovation of certain buildings'' (I6).*

Joint research activities with international partners were regarded as helpful *“to maintain our research potential. So that the researchers (…) would not leave the country (…), but rather be up to date with the leaders of their scientific field” (I10).* Participants saw the *“academic mobility of students and teachers” (S233_F)* as *“a great experience” (I1)* that enables students to learn in a peaceful atmosphere and teachers *“to see the best practices and, having returned home, to implement new teaching methods” (I1).* However, there were also concerns that the “*most experienced” (I7)* teachers might stay abroad*.* Also, participants envisioned more international collaborations for *“creating simulation centers” (I1)*, implementing *“Dual Master’s Degree Programs” (I5)* or having “*guest lectures, even if they were offered online” (I6).*

Participants emphasized the importance of interpersonal aspects of such international cooperation: *“Of course, it is important that we feel that we are not alone (…) when you experience something first-hand—the shelling and what that entails, the soldiers being killed, our population and our kids being killed—that constant stress, constant anxiety -. And to continue working under those conditions—no European, no other person [outside the situation] can understand that. Therefore, maintaining those contacts has significance in terms of practice and research, but also in terms of maintaining cultural ties, maintaining interpersonal, individual ties (…) one individual can have a major impact.” (I10).*

#### Quality of education

A deterioration of the quality of education negatively affects (a) motivation and (b) learning, leading to (c) less qualified graduates.

Participants described a change in student motivation in two directions: Students wanted *“more practice, working directly in the hospital, with patients" (S73_S)* as they are motivated to support their country. However, *“due to teaching being mostly remote and not always synchronous, [students’] motivation still gradually spirals down” (I6).* Therefore, teachers often had to *“give motivational talks to support students” (S132_F).*

While some participants believed that the quality of education *“has not deteriorated” (I8),* others were concerned that *“the consequences [of remote teaching] [would] be negative in terms of obtaining and assimilating knowledge” (S187_S).* Also, they anticipated that the limited access to patients and simulation centers would *“impair [students'] communication skills” (S247_F)* and exacerbate their lack of practical skills that already existed due to the COVID-19 pandemic: *“These periods will, no doubt, find their reflection on the quality of those students’ training, especially (…) the students who were in their fifth and sixth years of study.” (I4)*. To support students, *“the procedure for conducting tests” (S229_F)* was simplified, which potentially leads to *“a decrease in the qualification of doctors, because they took away the minimum passing score and changed the form of testing” (S134_S)*. While some students expected that the lack of practical training could *“be corrected by our own efforts now or during the internship” (S73_S)* or *“do not see any critical problems” (S133_S)* for their professional life, others were concerned that *“it will be more difficult to "acclimatize" during the internship” (S190_S)* or even to find one *“because after online training they do not want to hire me” (S285_S).*

#### Prospects of future professionals

This theme covers expected effects on students' professional lives in the future with (a) emphasis on war-related specialties, (b) fewer workplaces and difficult working environments for future professionals, and (c) pressure on the healthcare system.

Participants observed an emphasis on war-related specialties such as *“plastic surgery and prosthetic medicine” (S211_S) or “military surgeons”* (S64_S)*.* Some stressed the *“uniqueness of our experience”* (I5) which could be shared with other countries in the future. Also, they expected an *“increase in the number of patients requiring long-term physical and psychological rehabilitation” (S233_F)*, leading to a shift towards these specialties.

Participants expected a ***“****reduction of workplaces*” (S64_S) and a difficult working environment for future professionals since *“many health care facilities are destroyed” (S73_S). “The need for doctors will increase” (S13_S)* due to the war but at the same time there is an expected decrease in the number of physicians due to migration, burnout, or death on the frontlines, resulting in an *“increase in the amount of work” (S222_S)* in the future. Students feared that they might have to *“work in cities where large-scale hostilities are taking place, and (…) make a lot of efforts to rebuild hospitals and restoring the normal state” (S78_S)* or that *“it will be necessary to go to the front” (S111_S).*

There was *“a very powerful load on the health care system” (S4_S*) due to the loss of facilities, material, and healthcare professionals resulting in a *“deterioration in the quality of care” (S17_F).* Funding programs that were initiated years ago to improve *“the healthcare system of Ukraine will [now] have to be invested in the restoration of the system”* (S262_S). Despite these negative effects, participants remained hopeful: *“This cohesion and purposefulness demonstrated not only by doctors but also by the authorities (…) gives us hope that in the post-war period, all this will remain” (I7)* and that *"the war [will] be an impetus to improve medicine in Ukraine” (S93_S)*.

## Discussion

Our study shows that the quality of medical education in Ukraine is threatened by several war-related factors, mainly the disruption of teaching, financial restrictions, increased workload, and mental stress, all of which have a negative impact on students’ learning. Non-war-related general needs such as modern equipment or more practical training in the study programs exacerbate these challenges.

Our findings are largely consistent with those of the scoping review by Dobiesz et al. [21] on barriers and targeted interventions to sustain healthcare education during wartime. We found similar topics, such as threat to safety, loss of resources, structural changes in curricula, and use of online resources. However, due to the broader scope of our study, which included the perspective of faculty and staff, we put our findings into other themes. For example, as in Dobiesz et al., our participants reported a loss of students. This then led to a budgetary loss for schools which we categorized as “financial restrictions”, which is different from Dobiesz et al. who categorized the loss of students as an issue of “personnel”.

Beyond that, we identified the theme of prospects of future professionals with an anticipated increase in workload and worse working conditions. This is in line with recent publications about the situation of medical students inside and outside Ukraine [16, 36]; the students not only suffer from the current circumstances but also face an uncertain future. However, in contrast to recent studies in Iraq [22, 26] we did not identify a tendency among students to plan to leave the country. This might be because we collected data eight months after the war began, so the situation was still new and students might still have been hopeful that the war would end soon, whereas students in Iraq had already experienced years of armed conflict.

Participants described remote teaching as a proven measure used when teaching was disrupted, but consistent with recent literature the availability of internet, appropriate equipment, or access to platforms remained challenges [37–39].

In general, despite our efforts, we received very few responses from schools in the occupied zone. Thus, the measures described by the participants to continue and sustain teaching are probably mostly applicable to less affected regions, and we must assume that maintaining medical education in the occupied zone is even more challenging. Since the medical schools in the western cities have taken in many students who have migrated from the more affected regions, the situation is tense, with an unstable balance of students that is subject to constant change.

A major strength of this study is the triangulation of investigators, of methods (i.e., interviews and surveys), and of data sources (i.e., faculty leaders, faculty, staff, and students), that enabled us to “gain multiple perspectives and validation of data” [40].

While the data of most studies in the field of medical education during war have been collected retrospectively or after several years of ongoing conflict [24, 19], we identified restrictions, needs, and expectations during the first year of the war. To combine these approaches, we recommend conducting a longitudinal study across healthcare professions to examine how restrictions, needs, expectations, and perspectives change during and after a war. The themes we identify can serve as a valuable framework for implementing such a study, which could also investigate whether anticipated consequences were realized.

We are aware that our study has several limitations. First, we focused our sampling strategy on key stakeholders in medical education and did not specifically target other healthcare professions. Involving all healthcare professions might broaden the perspectives and new themes might even evolve. Second, as the situation in Ukraine has been constantly changing, our findings might be influenced by events at the time of data collection. For example, on the day we sent out the survey invitations, Russia started attacking the energy infrastructure across Ukraine [35] and consequently we identified stable electricity as an important need. Third, due to the translation of the interviews and survey responses into English, the meaning of participants’ statements may not have been fully reflected. However, we tried to minimize this effect, by employing an experienced Ukrainian researcher, double checking the translations, and discussing uncertainties in the team.

## Conclusions

In summary, we conclude that the majority of medical schools across Ukraine, including faculty, staff, and students, are facing various challenges to maintain medical education during the war. Although international partners and programs have supported schools in different ways, they could not compensate for all needs and the sustainability of the students’ education is still threatened. Therefore, our findings hopefully will allow future programs and collaborations to be better adapted to Ukraine’s specific needs, especially, by addressing the lack of bedside teaching and practical training. Regardless of the current war, participants have expressed the need to continue the reform process of medical education in Ukraine, which could be supported by international cooperation in several ways. For example, consultations with international partners could support the establishment of university hospitals or initiate new models of cooperation between medical schools and hospitals.

Also, researchers and educators outside of Ukraine can contribute by initiating joint research projects or providing train-the-trainer courses to support the advancement of medical education towards more case-based and learner-centered teaching approaches. In comparison to traditional teaching methods, these have been proven to be more effective in acquiring factual and practical knowledge and in supporting medical students in self-regulated learning [41–43]. A recent study from Japan showed that a learner-centered approach even stimulated self-regulated learning in students who were strongly accustomed to teacher-centered methods [44]. Especially in times when teaching is disrupted or there are not enough teachers available, self-regulated learning can be a helpful tool for students that contributes to maintain education. Medical schools in Ukraine welcome additional and flexible mobility programs with international partner schools, so that their students can fill teaching gaps, such as bedside teaching, to maintain the quality of education. Mobility programs can also help those medical schools that have been severely impacted by disruption of teaching or that have accepted many students from the more affected regions, by temporarily reducing the number of students.

These and other collaborations could be established through the European funding schema, such as international staff and student mobility programs, Erasmus+ higher education cooperation partnerships, or capacity building grants [45].

### Supplementary Information

**Additional file 1.**

## Data Availability

The datasets generated during and/or analyzed during the current study are not publicly available due to their qualitative nature but are available from the corresponding author on reasonable request.

## Footnotes

1 *Participants’ quotations are referenced as: I = interview, S = survey, _F = faculty and staff, _S = student.
