# Supplementary material for: Medical education in times of war: a mixed-methods needs analysis at Ukrainian medical schools
Source: BMC Med Educ. 2023 Oct 26;23:804. doi: 10.1186/s12909-023-04768-2 (PMC10605485; doi:10.1186/s12909-023-04768-2)
Supplement: Supplementary file 1 — Additional file 1. [file 12909_2023_4768_MOESM1_ESM.docx]

Additional file 1: Appendix 1

| **Survey for faculty and staff** | | | |
| --- | --- | --- | --- |
| **Question** | | **Answer options** | **# Responses (%)** |
| Please indicate your age: | | Number | 49 (100%) |
| Please indicate your sex: | | Female, Male, Other | 49 (100%) |
| What is your country of origin? | | Ukraine, Other | 49 (100%) |
| In which school and city do you work? | | Free text | 45 (92%) |
| What educational program do you relate mostly to? | | Medicine, Pediatrics, Other | 49 (100%) |
| What is your highest educational level and/or academic title? | | Junior Specialist, Specialist, Bachelor, Master, Ph.D./Doctoral degree, Postdoc, Associate/Assistant Professor, Professor, None, Other | 49 (100%) |
| How would you describe your primary role/roles at your school? | | Teacher of basic sciences, Teacher of clinical department, Administration of educational institution, Head of department, Dean, Deputy dean, Researcher, Other | 49 (100%) |
| How many years of work experience in healthcare education (excluding years of undergraduate study) do you have? | | Number | 49 (100%) |
| Has the region where you work been an active combat zone so far? | | Yes / No | 46 (94%) |
| Did you have to flee from home because of the war? | | Yes / No | 46 (94%) |
| If yes: | When and where did you go? Where are you living at the moment? | Free text | 9 (18%) |
| Are there any restrictions or changes to teaching at your school due to the war? | | Yes / No | 49 (100%) |
| If yes: | | | |
|  | Please describe any restrictions or changes that come to your mind. | Free text | 37 (76%) |
|  | What measures have been taken so far to handle the situation? | Free text | 36 (73%) |
|  | Please describe the needs to improve your current teaching situation and/or for the teaching at your school in general? | Free text | 32 (65%) |
| What future implications (due to the war) do you expect for teaching at your school? | | Free text | 39 (80%) |
| What future implications (due to the war) do you expect for the students? | | Free text | 39 (80%) |
| What future implications (due to the war) do you expect for the healthcare system? | | Free text | 38 (78%) |
| In your opinion, what are the most important needs of healthcare schools in Ukraine? | | 1., 2., 3., other | 37 (76%) |
| Is there anything else (not war-related) that should be improved regarding the teaching at your school? | | Free text | 30 (61%) |
| Do you want to add a comment (e.g., about something not mentioned in the survey that you consider important, or something you heard of other healthcare schools? | | Free text | 8 (16%) |
| Which of these teaching methods and forms have you applied online so far? | | Lectures, Seminars, Workshops, Case-based learning, Problem-based learning, Learning- Management-Systems (e.g., Moodle), Game-based learning (e.g., Kahoot!), Screencasts /videos, None, Other | 39 (80%) |

| **Survey for students** | | | |
| --- | --- | --- | --- |
| **Question** | | **Answer options** | **# Responses (%)** |
| Please indicate your age: | | Number | 177 (93%) |
| Please indicate your sex: | | Female, Male, Other | 190 (100%) |
| What is your country of origin? | | Ukraine, Other | 187 (98%) |
| In which school and city do you study? | | Free text | 172 (91%) |
| What educational program do you relate mostly to? | | Medicine, Pediatrics, Other | 190 (100%) |
| Your year of study: | | 1, 2, 3, 4, 5, 6, intern, doctoral/PhD studies, Other | 185 (97%) |
| What educational level are you applying for? | | Junior Specialist, Specialist, Bachelor, Master, Ph.D./ Doctoral degree, Other | 190 (100%) |
| How would you describe your primary role/roles at your school? | | Budget student (financed by the state), Contract student (self-payment), Exchange student, Part-time student, Tutor, Group leader, Other | 190 (100%) |
| Has the region where you study been an active combat zone so far? | | Yes / No | 141 (74%) |
| Did you have to flee from home because of the war? | | Yes / No | 144 (76%) |
| If yes: | When and where did you go? Where are you living now? | Free text | 31 (16%) |
| Are there any restrictions or changes to your study at your school due to the war? | | Yes / No | 173 (91%) |
| If yes: | | | |
|  | Please describe any restrictions or changes that come to your mind. | Free text | 101 (53%) |
|  | What measures have been taken so far to handle the situation? | Free text | 78 (41%) |
|  | Please describe the needs to improve your current study situation and/or the study at your school in general? | Free text | 76 (40%) |
| What future implications (due to the war) do you expect for your study? | | Free text | 117 (62%) |
| What future implications (due to the war) do you expect for your future professional activities? | | Free text | 112 (59%) |
| What future implications (due to the war) do you expect for the healthcare system? | | Free text | 112 (59%) |
| In your opinion, what are the most important needs of healthcare schools in Ukraine? | | 1., 2., 3., other | 113 (59%) |
| Is there anything else (not war-related) that should be improved regarding the study at your school? | | Free text | 74 (39%) |
| Do you want to add a comment (e.g., about something not mentioned in the survey that you consider important, or something you heard of other healthcare schools? | | Free text | 19 (10%) |
| In which of these teaching methods and forms have you participated online so far (during your study)? | | Lectures, Seminars, Workshops, Case-based learning, Problem-based learning, Learning- Management-Systems (e.g., Moodle), Game-based learning (e.g., Kahoot!), Screencasts /videos, None, Other | 142 (75%) |

Additional file 1: Appendix 2

| **Guideline for semi-structured interviews** |
| --- |
| Has the region where you work been an active combat zone? |
| Are there any restrictions or changes to teaching at your school due to the war? Please describe any restrictions or changes that come to your mind. |
| What measures have been taken so far to handle the situation? |
| What do you think are the most important needs of your school? |
| What future implications (due to the war) do you expect for teaching at your school? |
| What future implications (due to the war) do you expect for the students? |
| What future implications (due to the war) do you expect for the healthcare system? |
| What wishes or suggestions do you have for future cooperation and policy development, nationally and internationally? |

Additional file 1: Appendix 3

Table 1: Overview of participants’ schools and cities

| **School** | **City** | **# Interviews** | **# Faculty and staff** | **# Students** |
| --- | --- | --- | --- | --- |
| Cherkasy National University named after B. Khmelnytsky | Cherkasy | 0 | 1 | 8 |
| Bukovinian State Medical University | Chernivtsi | 1 | 11 | 41 |
| Dnipro State Medical University | Dnipro | 1 | 3 | 8 |
| Donetsk National Medical University | Donetsk | 1 | 0 | 0 |
| Ivano-Frankivsk National Medical University | Ivano-Frankivsk | 0 | 0 | 2 |
| Kharkiv National Medical University | Kharkiv | 1 | 3 | 2 |
| V. N. Karazin Kharkiv National University | Kharkiv | 0 | 0 | 1 |
| Bogomolets National Medical University | Kyiv | 1 | 0 | 4 |
| Taras Shevchenko National University of Kyiv | Kyiv | 0 | 11 | 18 |
| Shupyk National Healthcare University of Ukraine | Kyiv | 0 | 5 | 1 |
| -not explicitly stated- | Kyiv | 0 | 0 | 5 |
| Danylo Halytsky Lviv National Medical University | Lviv | 1 | 1 | 3 |
| Odessa National Medical University | Odessa | 1 | 0 | 0 |
| Luhansk State Medical University | Rivne | 0 | 1 | 0 |
| I. Ya. Horbachevsky Ternopil National Medical University | Ternopil | 1 | 2 | 0 |
| Uzhhorod National University | Uzhhorod | 0 | 1 | 0 |
| Pirogov National Medical University, Vinnytsia | Vinnytsia | 0 | 4 | 73 |
| Lesya Ukrainka Volyn National University of Vinnytsia | Vinnytsia | 0 | 1 | 0 |
| Zaporizhzhia State Medical University | Zaporizhzhia | 0 | 0 | 1 |
| Zhytomyr Medical Institute | Zhytomyr | 1 | 1 | 5 |
| Missing | - | 0 | 4 | 18 |
| **Total** |  | **9** | **49** | **190** |

# 
